# Molecular characterization and expression analysis of two peptidoglycan recognition proteins (CcPGRP5, CcPGRP6) in larvae ontogeny of common carp *Cyprinus carpio* L*.* and upon immune stimulation by bacteria

**DOI:** 10.1186/s12917-018-1744-1

**Published:** 2019-01-07

**Authors:** Fumiao Zhang, Shijuan Shan, Xiaoyang Xu, Yao Wang, Yonghuan Zhang, Miao Yin, Guiwen Yang

**Affiliations:** Shandong Provincial Key Laboratory of Animal Resistance Biology, College of Life Sciences, Shandong Normal University, No. 88 East Wenhua Road, Jinan, 250014 People’s Republic of China

**Keywords:** PGRP, Common carp, Larva, *Aeromonas hydrophila*

## Abstract

**Background:**

Although teleost fish developed acquired immunity firstly in evolution, innate immunity is still very important for them. Innate immunity depends on pattern recognition receptors (PRRs) to distinguish “self” and “non-self”, Peptidoglycan (PGN) recognition protein (PGRP) is one of the receptors and it can bind to multiple components of bacterial envelope.

**Results:**

We report the cloning and expression analysis of two PGRPs (*Ccpgrp5* and *Ccpgrp6*) from common carp (*Cyprinus carpio* L). The *Ccpgrp5* gene encodes a protein of 199 amino acid (aa) with PGRP domain, Ami_2 domain and four Zn^2+^ binding sites required for amidase activity, but without signal peptide and transmembrane domain. The *Ccpgrp6* gene encodes a protein of 446 aa with PGRP domain, Ami_2 domain, signal peptide, five Zn^2+^ binding sites required for amidase activity and two sites for N-glycosylation. The phylogenetic analysis revealed that the CcPGRP5 and CcPGRP6 are closely related to *Ctenopharyngodon idella* and *Danio rerio*. *Ccpgrp5* and *Ccpgrp6* were expressed in all tissues examined including liver, spleen, muscle, oral epithelium, head kidney, gill, skin, gonad, brain, foregut and hindgut and showed different distribution characteristics. During the embryonic and early larval developmental stages of common carp, *Ccpgrp6* was detected to be highly expressed at 10 days post fertilization(dpf) and 36 dpf, while *Ccpgrp5* were hardly detected using Real-time quantitative PCR. After being challenged with *Aeromonas hydrophila*, *Ccpgrp5* in adult common carp was induced and up-regulated in all the tissues, especially in gill and spleen, but not in head kidney, while *Ccpgrp6* was up-regulated in all the tissues, especially in liver, head kidney and gill. The varied expression profiling of *Ccpgrp5* and *Ccpgrp6* indicated they had different roles in the host immune response.

**Conclusions:**

These results indicated the two PGRPs, especially *Ccpgrp6*, played an important role in the immune defense of common carp during larva development and against *Aeromonas hydrophila*, providing insight to further exploration of protecting fish against bacteria infectious disease.

## Background

Innate immune system offers germline-encoded immediate protection for the host from pathogen infections and has retained its antimicrobial effectiveness for millions of years in all multicellular organisms [1]. Although fish is the first vertebrate to develop adaptive immunity, they still defend against pathogens depending on the innate immune mechanism primarily until their adaptive immune system has developed, especially their eggs and embryos which are laid and develop in water [2]. Even in adult fish, the function of innate immunity are still irreplaceable in their life [3, 4].

Peptidoglycan recognition protein (PGRP) is one of pattern recognition receptors (PRRs) and it can recognize common component of bacterial cell wall such as peptidoglycan (PGN), lipoteichonic acid (LTA), and lipopolysaccharide (LPS) [5]. So it is possible for immune cells to discriminate the pathogens from the host cells.

PGRPs are conserved in most animal species from insects to mammals, containing the Ami_2 domain and PGRP domain [5]. The Ami_2 domain is homologous to type II amidase of bacteria and phage lysozyme, which enable PGRP to interact with pathogens and kill the invading pathogens directly with Zn^2+^ [6]. The first peptidoglycan recognition protein was found in the blood of *Bombyx mori*, which can bind to PGN without Ca^2+^, activate prophenoloxidase cascade and induce humoral melanization [7, 8]. More than 100 peptidoglycan recognition proteins have been found in all species at present, which are expressed in varied tissues, notably in tissues of the immune system such as bone marrow and peripheral neutrophils of bovine [9], bone marrow and spleen of porcine [10], liver, head kidney and spleen of grass carp [11], spleen and liver of Chinese giant salamander [12]. On one hand PGRPs have amidase activity to hydrolyze the lactyl-amide bond between MurNAc and 1-Ala in the PGN and on the other hand trigger the Toll or Immune deficiency (Imd) signal transduction pathway to generate antimicrobial peptides [5]. As to vertebrate PGRPs, there are four paralogs in mammals. They usually presented disulphide-linked homo and heterodimers with both recognition and effector functions [13]. PGLYRP-2 secreted from liver into blood is an N-acetylmuramoyl-L-alanine amidase and PGLYRP-1, PGLYRP-3 and PGLYRP-4 are also bactericidal or bacteriostatic proteins which were different from known vertebrate antimicrobial [14]. Recent studies indicated that PGRP could induce oxidative, thiol, and metal stress responses simultaneously in bacteria through three independent pathways [15].

Among fish species, PGRP molecules were found in zebra fish (*Danio rerio*) [16, 17], rockfish (*Sebastes schlegeli*) [18], rock bream (*Oplegnathus fasciatus*) [19], large yellow croaker (*Pseudosciaena crocea*) [20], channel catfish (*Ictalurus punctatus*) [21], green-spotted pufferfish (*Tetraodon nigroviridis*) [16], grass carp (*Ctenopharyngodon idella*) [11, 22, 23], rainbow trout (*Oncorhynchus mykiss*) [24, 25], turbot(*Scophthalmus maximus*) [26], tongue sole (*Cynoglossus semilaevis*) [27], and red drum (*Sciaenops ocellatus*) [28]. The sequence of PGRP5 and PGRP6 gene are also cloned from common carp (*Cyprinus carpio*). Among them, three types of PGRP were identified including PGRP2, PGRP5 and PGRP6, with only PGRP-2 homologous to mammal PGLYRP2, while PGRP5 and PGRP6 are found only in teleost fish [21]. *Ccpgrp5* and *Ccpgrp6* of common carp *are* short PGRP and long PGRP molecules respectively, but the response and expression of *Ccpgrp5* and *Ccpgrp6* are not known. In other fish, the PGRPs take the important role in resistance of bacteria. Firstly, previous studies demonstrated that teleost fish PGRP had both amidase and bactericidal activities in one molecule, including SsPGRP-L1 and SsPGRP-L2 from the rock fish, PGLYRP-2, PGLYRP-5 and PGLYRP-6 from zebrafish [18]. Secondly, fish PGRP played immunomodulatory roles in the immune response to bacteria, such as OmPGRP-L1 and OmPGRP-L2 from rainbow trout, PGRP-SC2 from rock bream. As for the distribution of fish PGRPs, they were expressed ubiquitously in many tissues, and their constitutive expression levels were higher in classical immune tissues than in mucosal tissues. Furthermore, the up-regulation of PGRP could be induced by bacterial challenge. For instance, SmPGRP2 from turbot induced by *Streptococcus iniae* and *Vibrio anguillarum*, and RbPGRP-SC2 from rock bream induced by *Edwardsiella piscicida*, *Streptococcus iniae*. However, only a few studies have investigated the expression profiling of PGRPs in larvae ontogeny and its response upon immune stimulation by *Aeromonas hydrophila* in vertebrates including teleost fish.

Carp is one of the most popular cultured fish in China and the diseases caused by *Aeromonas hydrophila* can do great harm to carp aquaculture. *Aeromonas hydrophila* is resistant to antibiotics attributed to the indiscriminate use of antibiotics in aquaculture and plasmid or horizontal gene transfer [29–31]. However, innate immune system offers germline-encoded immediate protection for the host from infections and has retained its antimicrobial effectiveness for millions of years with no frequent emergence of resistant strains. Here we cloned two carp PGRPs, named as *Ccpgrp5* and *Ccpgrp6*, and showed their expression profiling in larvae ontogeny, normal adult tissues and adults tissues exposed to *Aeromonas hydrophila*.

## Results

### cDNA sequence of *Ccpgrp5* and *Ccpgrp6*

**Fig. 1:**
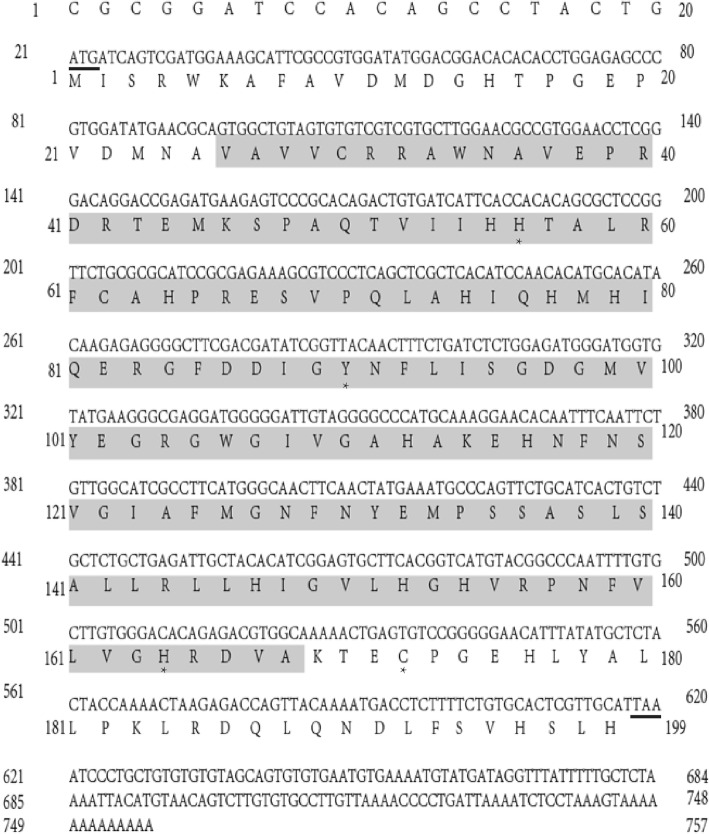
Nucleotide and deduced amino acid sequences of common carp peptidoglycan recognition protein5, *Ccpgrp5*. The translation start codon ATG and the termination codon TAA are indicated under line. The PGRP domain is shown in grey (26-168aa). Zn^2+^ binding sites (H56, Y90, H164, C172) are marked with asterisk

**Fig. 2:**
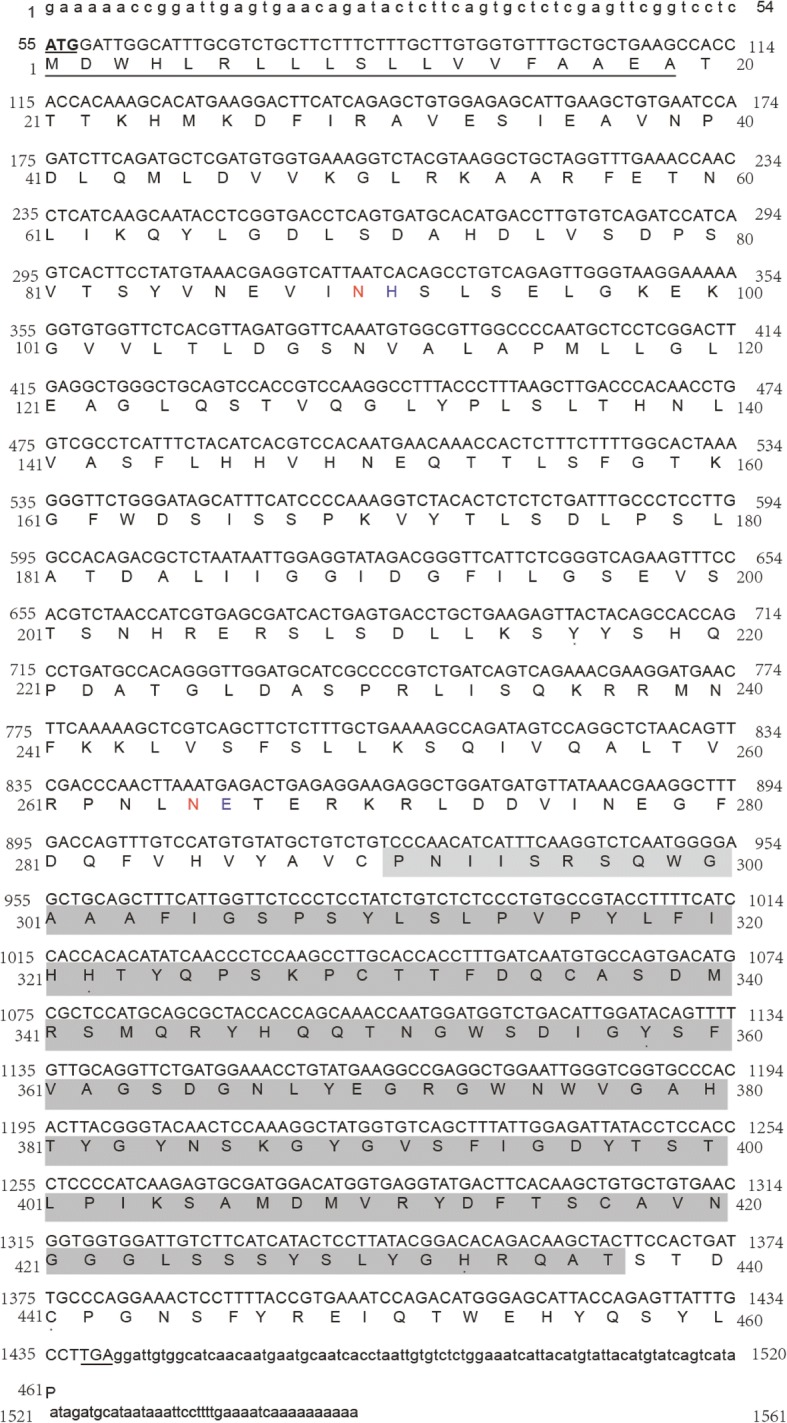
Nucleotide and deduced amino acid sequences of common carp peptidoglycan recognition protein 6, *Ccpgrp6*. The translation start codon ATG and the termination codon TAA are indicated in dotted line. The sequence of signal peptide is indicated under line (1-19aa). The PGRP domain is shown in grey (291-437aa). Zn^2+^ binding sites (Y216, H322, Y358, H433, C441) are marked with asterisk. The predicted N-glycosylated sites are highlighted in red (Asparagines) and blue (Asn-Xaa-Ser/Thr sequences)

Using 3′- and 5’-RACE, we identified two PGRP candidates from the total RNA of common carp, the *Ccpgrp5* and the *Ccpgrp6*. The cDNA of *Ccpgrp5* (GenBank Accession number MF818332) is 757 bp in length including a 20 bp 5′-untranslated region (UTR), an 597 bp ORF and a 140 bp 3’-UTR, which encodes a protein of 199 amino acids with a predicted isoelectric point (PI) of 7.095 (Fig. 1). The sequence of CcPGRP5 shared 100% sequence identities and 96% query cover with previous PGRP5 sequence of *Cyprinus carpio* (KT224436). The full-length *Ccpgrp6* cDNA (GenBank accession number MG272264) amplified from the spleen of common carp was 1561 bp in length, including a 54 bp 5′-untranslated region (UTR), an open reading frame (ORF) of 1383 bp and a 124 bp 3’-UTR. The ORF of *Ccpgrp6* encoded a putative protein of 461 amino acids with a predicted isoelectric point (PI) of 6.467 (Fig. 2).The sequence of CcPGRP6 shared 98% sequence identities and 99% query cover with previous PGRP6 sequence of *Cyprinus carpio* (KU642466).

### Homology alignment and phylogenetic analysis

Sequence analysis of CcPGRP5 and CcPGRP6 indicated that they were highly homologous to PGRPs from other species, especially at their C-terminals. As a short PGRP, CcPGRP5 is highly homologous to grass carp (*Ctenopharyngodon idella*) PGRP5 (88%) and zebra fish (*Danio rerio*) PGRP5 (84%). As a member of long PGRP, the amino acid sequence of CcPGRP6 are homologous to grass carp (*Ctenopharyngodon idella*) PGRP6 (84%) and zebrafish (*Danio rerio*) PGRP6 (73%).

**Fig. 3:**
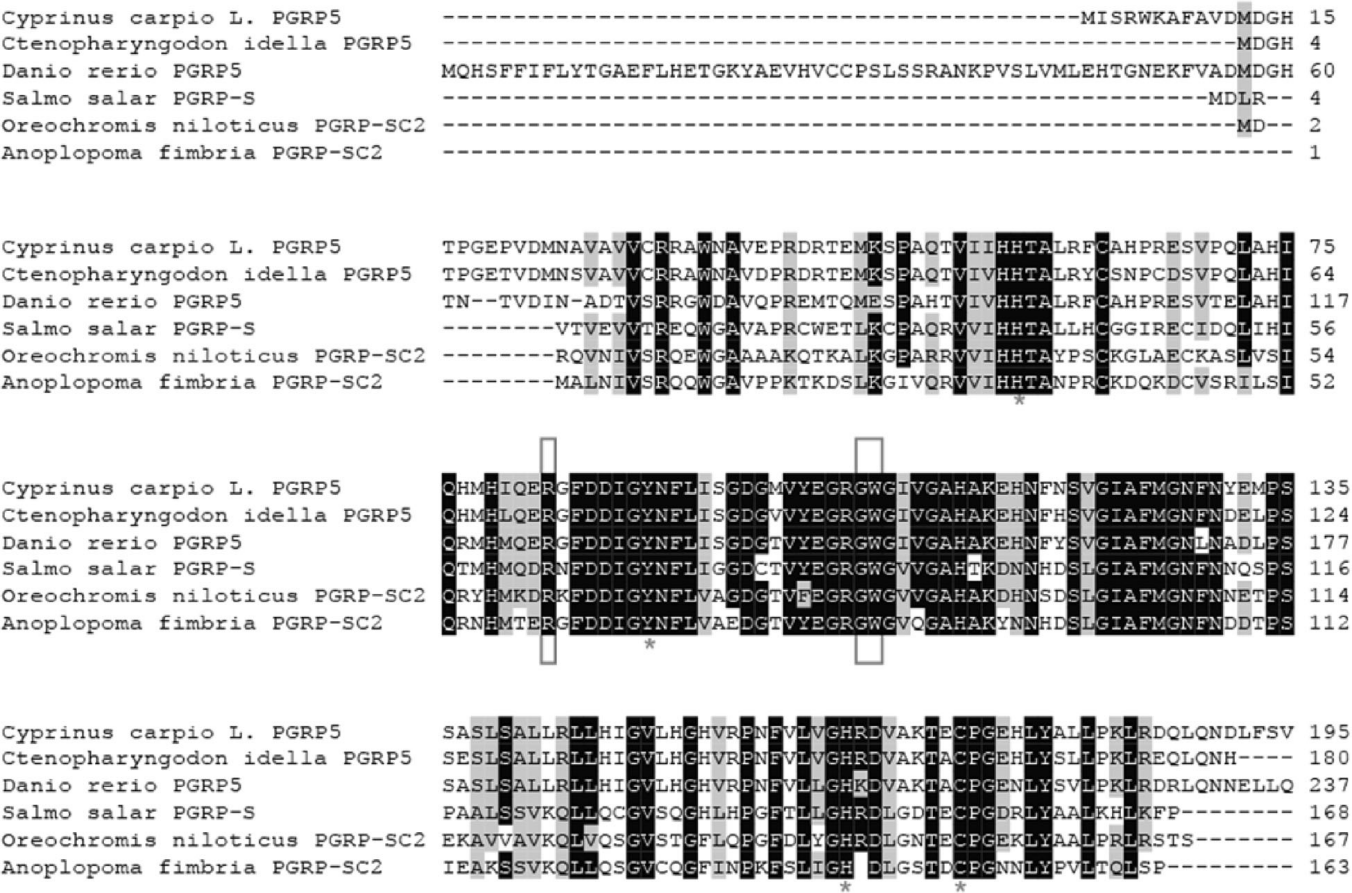
Multiple sequence alignment of the deduced CcPGRP5 with that of other five fish species. The amino acid sequences of PGRP5/PGRP-SC in *Cyprinus carpio* (MF818332), *Ctenopharyngodon idella* (AFE8096), *Danio rerio* (NP_001037786), *Salmo salar* (BT049722), *Oreochromis niloticus* (XP_003441739) and *Anoplopoma fimbria*(ACQ58764) are deduced from cDNA. Residues conserved in all species are shaded in black. The right number represent the amino acid position in the corresponding species. The conserved amino acid residues (R83, G105 and W106) shown in box are sites for recognizing DAP-type peptidoglycan specifically. Zn^2+^ binding sites (H56, Y90, H164, C172) are marked with asterisk

**Fig. 4:**
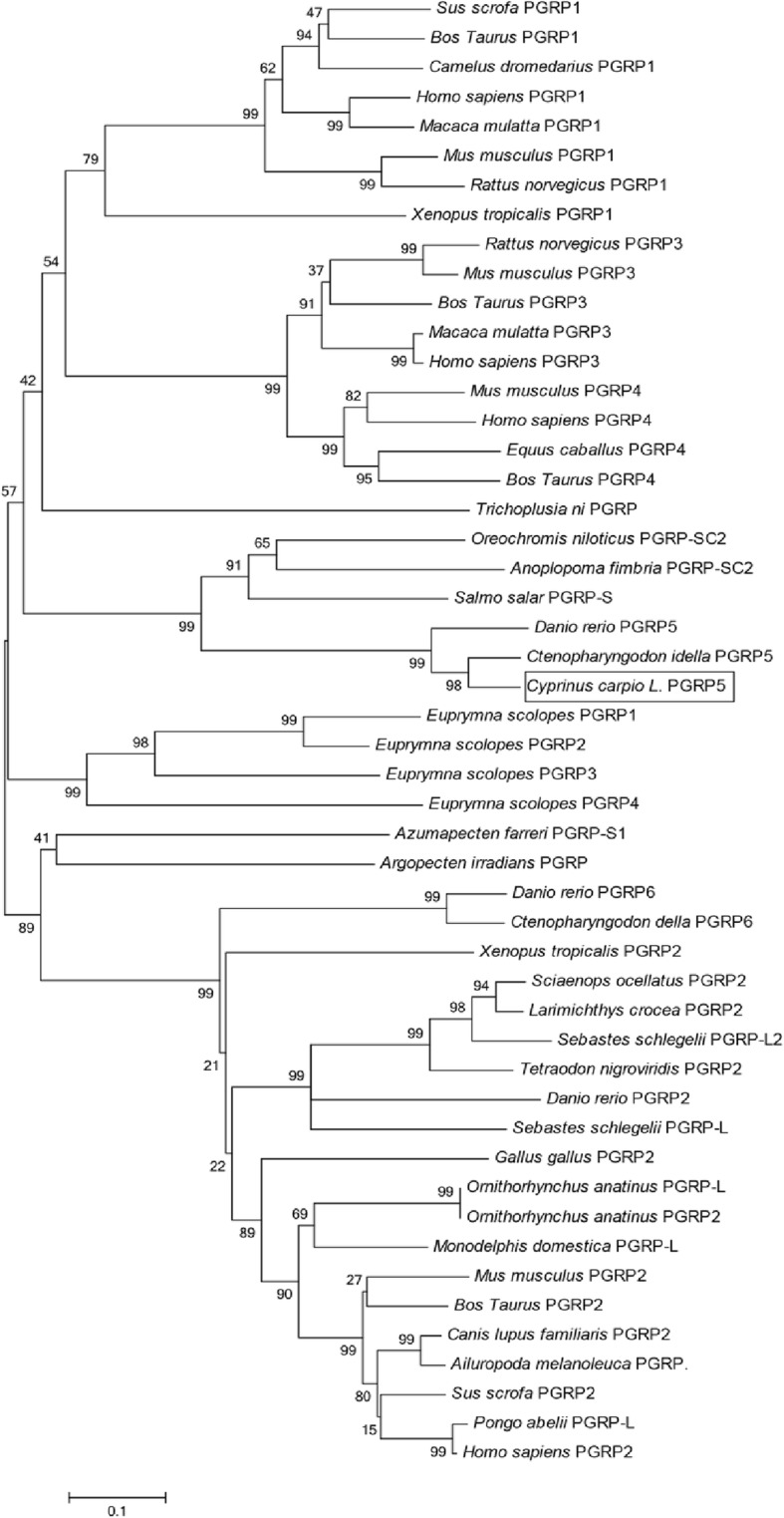
Phylogenetic tree analysis of CcPGRP5 with all known PGRPs from the other species. Phylogenetic tree was obtained from a CLUSTALW alignment and MEGA 6.0 Neighbor-Joining of 50 sequences. The bar indicated the distance

Sequence analysis indicated that CcPGRP5 had four Zn^2+^ binding sites (His56, Tyr90, His164, Cys172) for amidase activity and three conserved binding sites (Arg83, Gly105, Trp106) for recognizing DAP-type peptidoglycan specifically (Figs. 1 and 3). Analysis using SignalP 4.0 server indicate that CcPGRP5 has no signal peptide and no transmembrane domain to function in cytosol. Using the SMART program, the CcPGRP5 was found to comprise a PGRP domain (26–168 aa) and an Ami-2 domain (37–174 aa). To investigate the evolutionary relationships of CcPGRP5 with that of other species, a phylogenetic tree was constructed. The results showed that CcPGRP5 is most similar to grass carp (*Ctenopharyngodon idella*) PGRP5 and Zebrafish (*Danio rerio)* PGRP5 (Fig. 4).

**Fig. 5:**
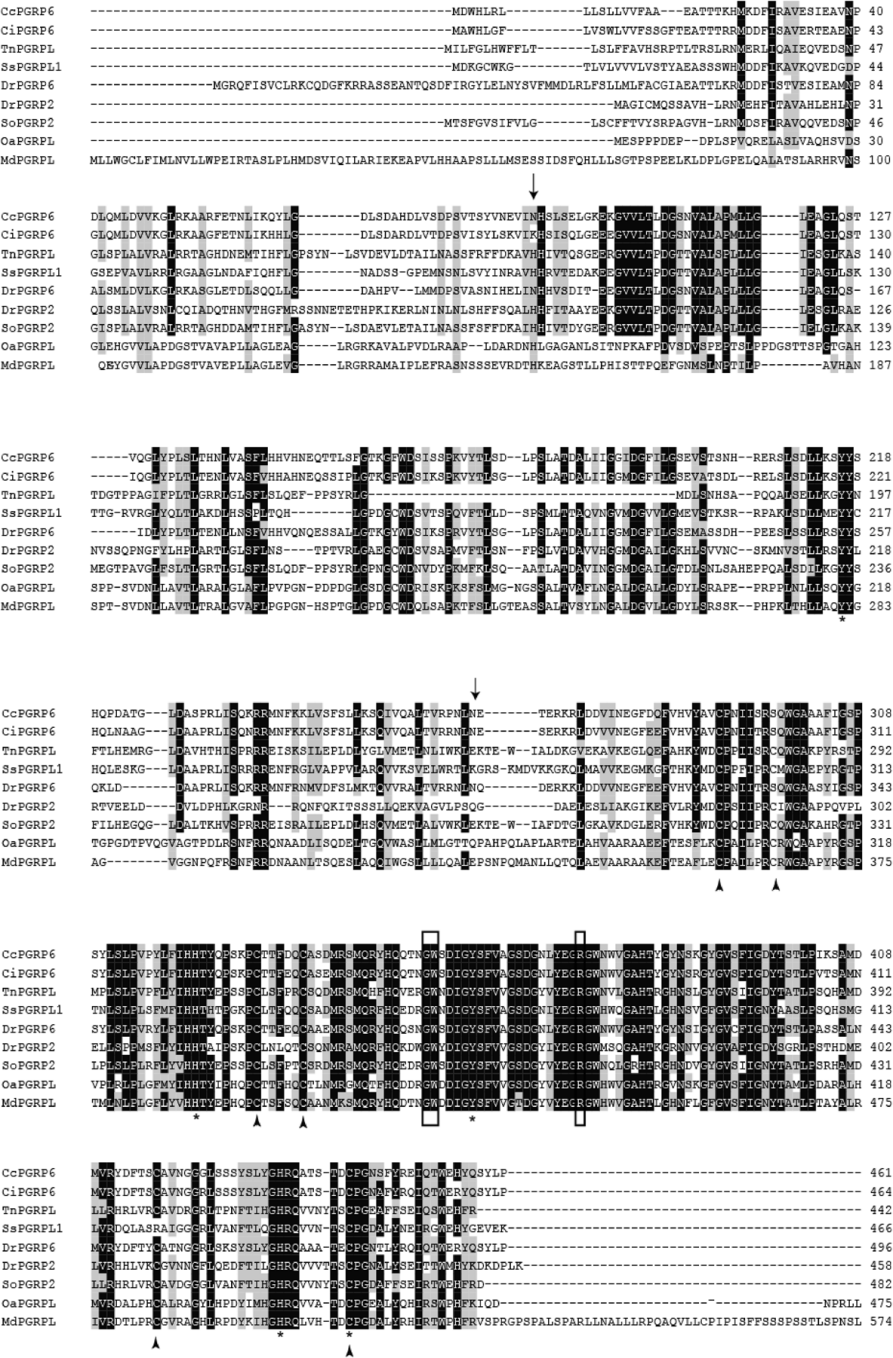
Multiple sequence alignment of the deduced CcPGRP6 with that of other five fish species. The amino acid sequences of PGRP6/PGRP2/PGRP-L/L1in *Cyprinus carpio* (MG272264), *Ctenopharyngodon idella* (ADL411866), *Tetraodon nigroviridis* (CAG06114), *Sebastes schlegelii* (ADC93708), *Danio rerio* (NP_001038631, NP_001038687), *Sciaenop socellatus* (GU126381), *Ornithorhynchus anatinus* (XP-001506175) and *Monodelphis domestica* (XP-001363587) are deduced from cDNA. Residues conserved in most species are shaded in black. The right number represent the amino acid position in the corresponding species. The conserved amino acid residues (G452, W453 and R472) shown in box are sites for recognizing DAP-type peptidoglycan specifically. Zn^2+^ binding sites (Y216, H322, Y358, H433, C441) are marked with asterisk.Conserved cysteine residues to form disulfide bonds are marked with arrow

**Fig. 6:**
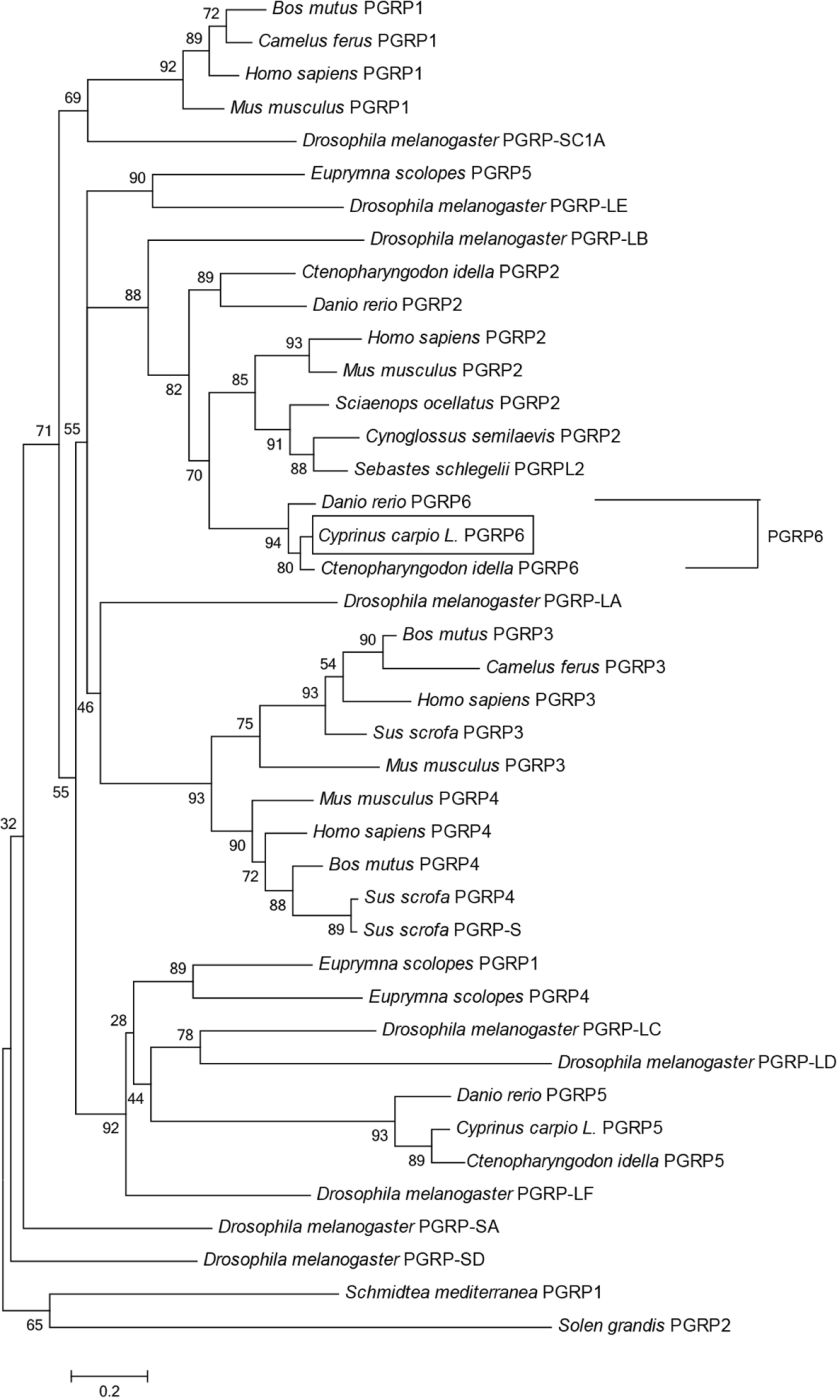
Phylogenetic tree analysis of CcPGRP6 with all known PGRPs from the other species. Phylogenetic tree was obtained from a CLUSTALW alignment and MEGA 6.0 Neighbor-Joining of 50 sequences. The bar indicated the distance

Multiple sequences alignment with other long PGRPs, including zebrafish (*Danio rerio*) PGRP6 and PGRP2, grass carp (*Ctenopharyngodon idella*) PGRP6, opossu (*Monodelphis domestica)* PGRP1, platypus (*Ornithorhynchus anatinus*) PGRP-L, red drum (*Sciaenop socellatus*) PGRP-2, rockfish (*Sebastes schlegelii*) PGRP-L2, and puffer fish (*Tetraoden migroviridis*) PGRP-L showed that the C-terminal of PGRP, which was characteristic of amidase activity, were highly conserved. However, N-terminals in these species did not show significant homologous. Sequence analysis indicated that CcPGRP6 has five Zn^2+^ binding sites (Tyr216, His322, Tyr358, His433, Cys441) for amidase activity, three conserved binding sites (Gly452, Trp453, Arg472) for recognizing DAP-type peptidoglycan specifically, and conserved Cys residues that form disulfide bonds (Fig. 5). CcPGRP6 contains a PGRP domain (291–437 aa) and an Ami_2 domain (303–443 aa). Analysis using SignalP 4.0 server indicate that CcPGRP6 may function as a secreted protein because it has predicted signal peptides composed of 19 amino acid residues (1–19 aa) and no transmembrane domain. To investigate the evolutionary relationships of CcPGRP6 with other species, a phylogenetic tree was constructed. The results showed that CcPGRP6 is clustered closely with the PGRP6 of grass carp (*Ctenopharyngodon idella*) and Zebrafish (*Danio rerio)* (Fig. 6).

### Constitutive expression of the *Ccpgrp5* and *Ccpgrp6*

**Fig. 7:**
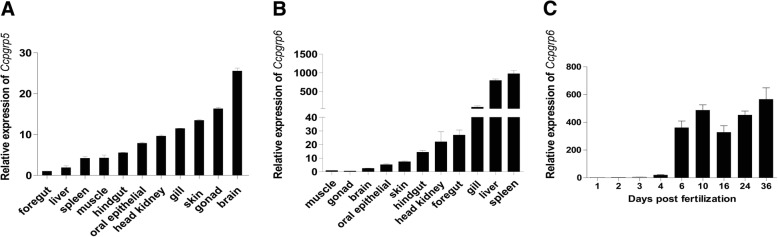
Tissue expression profile of *Ccpgrp5* and *Ccpgrp6*. **a** *Ccpgrp5* transcripts in the brain, gonad, skin, gill, head kidney, oral epithelium, hindgut, muscle, spleen, liver and foregut of common carp are detected by Real-time quantitative PCR. **b** *Ccpgrp6* transcripts in the spleen, liver, gill, foregut, head kidney, hindgut, skin, oral epithelium, brain, gonad and muscle of common carp are detected by Real-time quantitative PCR. **c** *Ccpgrp6* gene expression during the developement of common carp larvae between 1 and 36 days post fertilazaiton. Amplification of S11 in each tissue is performed as an internal control. Data plotted were mean ± S.D. of three replicates, *n* = 3 (for **a** and **b**), Data plotted were mean ± S.D. of five replicates, *n* = 5 (for **c**)

To investigate the tissue-dependent expression pattern, we performed qRT-PCR analysis using gene-specific primers for *Ccpgrp5* and *Ccpgrp6*. The expression of the *Ccpgrp5* and *Ccpgrp6* were detected in almost all examined tissues of normal adult carp with varied expression levels in different tissues. Expression of *Ccpgrp5* was found to be highest in the brain and gonad, and then in decreasingly order in skin, gill, head kidney, oral epithelium, hindgut, muscle, spleen, liver and foregut (Fig. 7a). In contrast, *Ccpgrp6* was strongly expressed in the spleen, liver and gill, but weakly expressed in the foregut, head kidney, skin, oral, brain, gonad and muscle (Fig. 7b).

Further study on the constitutive expression of the two *Ccpgrps* gene in embryo and early larvae of common carp from 1 to 36 days post fertilization (dpf) showed that though the expression of *Ccpgrp6* was hardly detected at 1 dpf and 2 dpf, *Ccpgrp6* was relatively highly expressed at 10 dpf and 36 dpf (Fig. 7c). However, the expression level of *Ccpgrp5* was too low to be detected in embryo and early larva of common carp by Real-time quantitative PCR.

### Expression profiles of *Ccpgrp5* and *Ccpgrp6* in common carp challenged by *A. hydrophila*

**Fig. 8:**
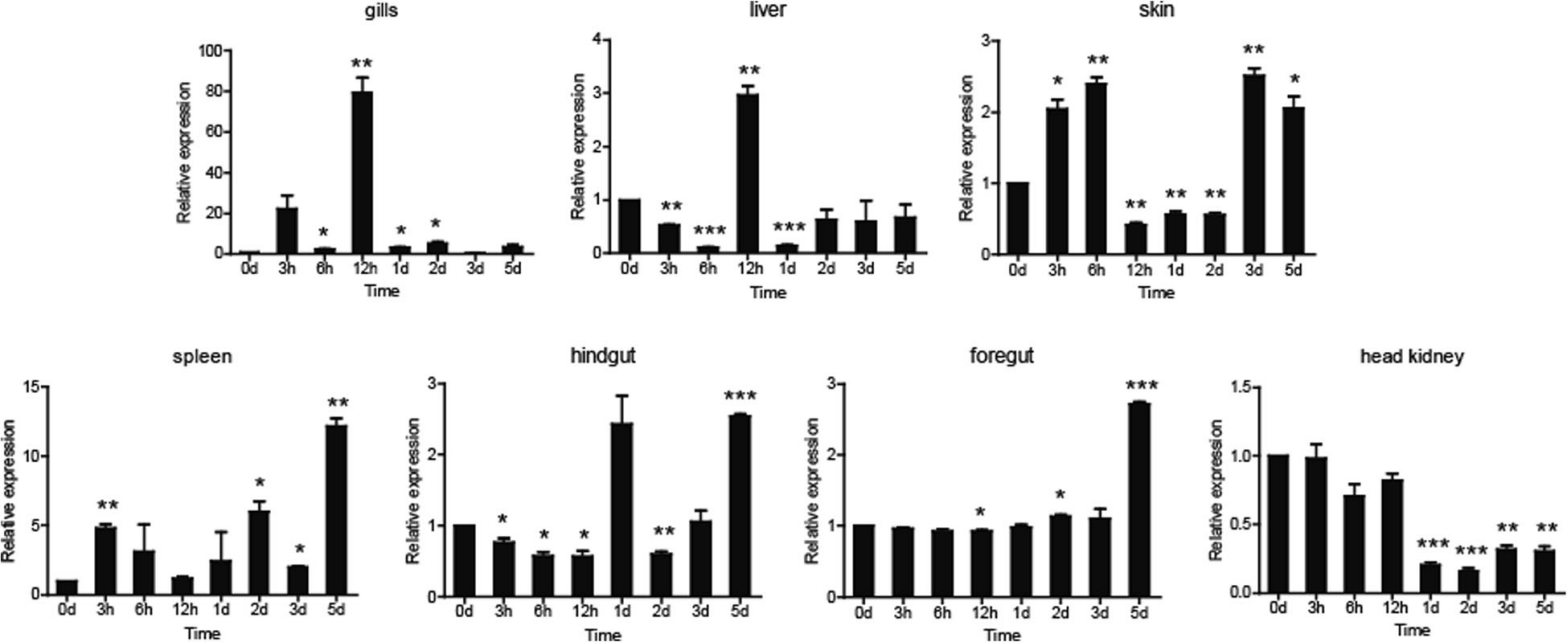
The relative expression of *Ccpgrp5* in common carp after i.p. injection with A.hydrophila. The time-dependent expression pattern of *Ccpgrp5* in the foregut, hindgut, liver, spleen and head kidney of common carp after infection. All the results are corrected by 40S ribosomal protein S11. Data are presented as a fold increase of the challenged group to the un-stimulated control group (not shown in the paragraph) are shown as the mean ± SEM (*n* = 3).*p < 0.05, **p < 0.01 or ***p < 0.001 versus unstimulated fish

**Fig. 9:**
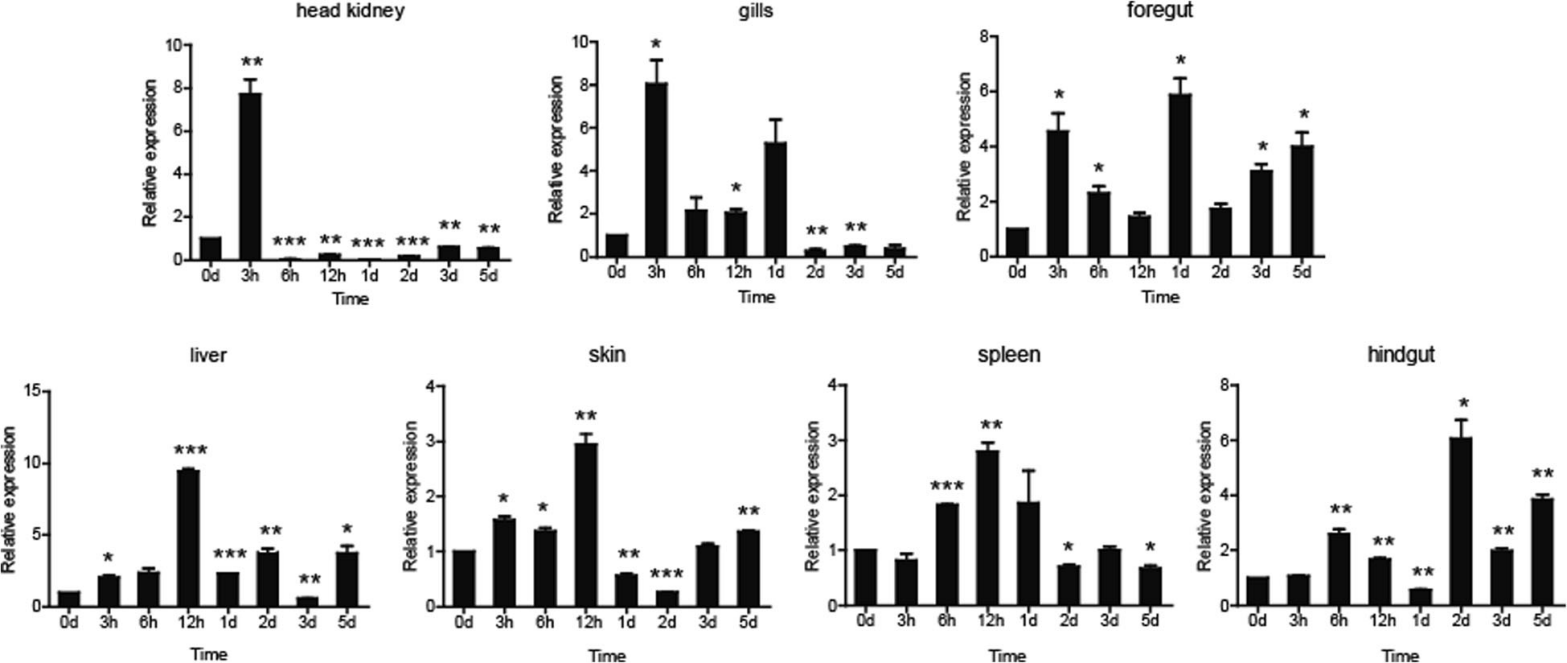
The relative expression of *Ccpgrp6* in common carp after i.p. injection with A.hydrophila. The time-dependent expression pattern of *Ccpgrp6* in the foregut, hindgut, liver, spleen and head kidney of common carp after infection. All the results are corrected by 40S ribosomal protein S11. Data are presented as a fold increase of the challenged group to the un-stimulated control group (not shown in the paragraph) are shown as the mean ± SEM (*n* = 3).*p < 0.05, **p < 0.01 or ***p < 0.001 versus unstimulated fish

Common carp was challenged with *A.hydrophila* in order to determine the expression profiles of PGRPs in response to bacterial infections. The two PGRPs exhibited distinctive tissue expression profiles. The expression of *Ccpgrp5* was significantly upregulated and reached the highest in the gill and liver at 12 hpi (hours post injection), with 80.5-fold and 2.9-fold higher than the control group respectively (*p* < 0.01), while the highest expression detected in spleen, foregut and hindgut appeared at 5 dpi (days post injection), with 12.1-fold in spleen, 2.7-fold in foregut and 2.5-fold in hindgut (*p* < 0.01 or *p* < 0.001 for all). Meanwhile, no significant fold increase were observed in head kidney and very minor increase in skin (about 2-fold), but decreased expression of *Ccpgrp5* in these two tissues at both 1dpi and 2dpi (Fig. 8). Compared with *Ccpgrp5*, the expression of *Ccpgrp6* in most tested tissues was different. In gills, the expression level of *Ccpgrp6* mRNA after *A.hydrophila* challenge was up-regulated highest with 8-fold at 3hpi, which is earlier than *Ccpgrp5*, but the fold change was moderate. In head kidney, the induced expression of *Ccpgrp6* was so obvious that their highest level reached 7.9-fold at 3hpi. The mRNA expression of *Ccpgrp6* after *A.hydrophila* challenge was detected highest in the other three tissues including liver, skin and spleen at 12 hpi, with 9.7-fold, 2.9-fold and 2.9-fold higher expression than the control group respectively (*p* < 0.01 or *p* < 0.001 for all). In foregut and hindgut, the expression of *Ccpgrp6* was induced for a longer period of time with higher fold change (about 6-fold) than that of CcPGRP5 (Fig. 9). The results indicated that *Ccpgrp6* might play a more important role in both system immune function and mucosal immune function of common carp challenged by *A.hydrophila.*

## Discussion

The structure of peptidoglycan recognition protein (PGRP) is highly conserved from invertebrates to vertebrates. Both CcPGRP5 and CcPGRP6 have conserved domain and amino acid residues in sequences which are related with their function. The study of common carp showed that both CcPGRP5 and CcPGRP6 have PGRP domain and Ami_2 domain. The Ami_2 domain at C terminal of most of PGRP is a type 2 amidase domain, which is homologous to the type 2 amidase of bacteriophages and bacteria. In addition, sequences alignment showed that Zn^2+^ binding sites existed in both CcPGRP5 (His56, Tyr90, His164 and Cys172) and CcPGRP6 (Tyr216, His322, Tyr358, His433, Cys441), which indicates that they may have Zn^2+^ dependent amidase activity. Furthermore, the conserved binding sites for specific recognition of DAP-type peptidoglycan also existed in CcPGRP5 (Arg83, Gly105, Trp106) and CcPGRP6 (Gly452, Trp453, Arg472). As we know, Dap-type peptidoglycans are components of many Gram-negative and some Gram-positive bacteria. Long PGRP, such as *Drosophila* membrane PGRP-LC, recognize Dap-type peptidoglycans and activates both the imd pathway and the proPO cascade [32]. Besides, CcPGRP6 may function as a secreted protein because it has a signal peptide and no transmembrane, while CcPGRP5 may be present in the cytosol due to the lack of signal peptide and transmembrane.

PGRP is usually expressed in blood cells, such as hemolymph of silkworm and arthropods, and neutrophils of mammals [8, 13]. However, the expression of PGRP in other tissues could also be detected, such as fat bodies of insects, gills, muscles, gonads, hepatopancreas of mollusks [33], liver, intestine, stomach in Amphibian and head kidney, spleen, gill, intestine, skin, liver of fish, Furthermore, PGRP of most these tissues demonstrated amidase activity. As for the tissue specific expression patterns of *Ccpgrp5* and *Ccpgrp6*, the two molecules are very different. Firstly, *Ccpgrp6* is substantial highly expressed in the immune related tissues such as spleen and liver, followed by the expression in gill and foregut which directly contacts pathogens, and then muscle and gonad etc. However, the highest expression of *Ccpgrp5* is in brain, followed by gonad, skin and gill, with the expression in liver and spleen being less than that of muscle, hindgut, oral epithelial and head kidney. The expression variance among different tissues is smaller for *Ccpgrp5* than that for *Ccpgrp6*. Brain is the main viral target tissues and the gonad used to transmit the virus vertically even in teleost. The study of European sea bass infected with viral nervous necrosis virus (VNNV) in the brain showed that upregulation of interferon (IFN) and different IFN-stimulated genes could be induced in the brain and the gonad. They thought the brain innate immune response is unable to clear the virus and pointed to the importance of gonad immunity to control the dissemination of VNNV to the progeny [34]. Demonstration of the constitutive expression of *Ccpgrp* in common carp tissues from this study indicated that the *Ccpgrp6* may play an important role in the basic immune protection of carp, while the role of *Ccpgrp5* may be completely different. The comparatively higher expression of *Ccpgrp5* in brain and gonad might indicated that this molecule may have important role in the function of these two tissues. Actually, Except for *Ccpgrp5*, some other immune-related gene including Rig-1 like receptor [35], Toll like receptor [36], XBP-1 [37] are all expressed in gonad. In mammals, It was widely accepted that the process of ovulation is similar to that of inflammation, many genes expressed in the gonad involved in the inflammatory response are also involved in the ovulation process.

*A. hydrophila* is a Gram-negtive bacterium. The *Ccpgrp5* and *Ccpgrp6* of carp challenged by *A.hydrophila* can be induced in different tissues for varied period of time. In previous studies, the PGRP5 in kidney cells of grass carp could be induced by pathogenic factors such as PGN, LTA and poly I:C [11]. In this experiment, although the expression level in gills and spleen of normal carp are not the highest compared with other tissues, the increase of *Ccpgrp5* expression in gills and spleens of carp after *A.hydrophila* challenge is more higher. It was nearly 80-fold increase in gills at 3 h post injection (hpi) and 12.1-fold in spleen at 12 hpi respectively. As to *Ccpgrp6*, the expression increase was not as high as that of *Ccpgrp5*. It was around 6-fold ~ 10-fold in head kidney, gills, foregut, liver and hind gut. However, it needs to be considered that the basic expression of *Ccpgrp6* is relatively higher in tissue of spleen, liver, gill and even foregut of normal carp, so the amount of induced production of *Ccpgrp6* during *A.hydrophila* stimulation is considerable.

Fish in aquatic environment are facing survival challenges from pathogenic microbes in the water environment. The immune function of adult fish plays an important role in defending against pathogenic bacteria. Due to the unknown involvement of CcPGRP for the establishment of immunity in the phase of egg and larvae in fish, this study examined the expression of two *Ccpgrp* in the early embryos of carp. The results showed that the *Ccpgrp5* expression could be hardly detected at 1,2,3,4 days post fertilization (dpf) and the expression of *Ccpgrp6* began to increase significantly at 6 dpf, then the expression level reached to the first peak at 10 dpf, followed by a slightly decrease at 16dpf and 24 dpf, and finally reached the highest expression level at 36dpf. The *Ccpgrp6* expression profile in larvae of common carp indicated that it might involve in the defense against pathogens in early development stage in aquatic environment. In contrast, *Ccpgrp5* could not be detected during the examined development stage. These results were different with what were reported for large yellow croaker and zebra fish. In large yellow croaker, PGRP2 expressed highly in unfertilized egg and kept at very low expression level during larvae [20]. In zebrafish, PGLYRP-2 is strongly expressed in the egg and both PGLYRP-2 and PGLYRP-5 are expressed in the developing embryo. On the contrary, PGLYRP-6 protein could not be detected in the eggs or in the early stages of development of zebrafish [16]. Due to the limited study of PGRP family in egg and larvae of fish, the variance of PGRP between different fish species needs further study.

## Conclusion

This study investigated the structure, evolutionary relationship and expression characteristics of two PGRP genes from common carp. Both *Ccpgrp5* and *Ccpgrp6* have the conserved PGRP domain and Ami_2 domain and they are highly homologous to the short and long PGPRs in all the vertebrates and invertebrates respectively. The constitutive expression of *Ccpgrp5* and *Ccpgrp6* in various tissues of adult carp and during early larval ontogeny implies their possible relevance to immune function of common carp and indicated the different role and activation pathway between the two CcPGRPs. Moreover, the up-regulated expression of *Ccpgrp5* and *Ccpgrp6* strongly indicates that they play a significant role in innate immune defense against bacteria.

## Methods

### Ethics statement

The protocol was approved by the Animal Experimental Ethics Committee of Shandong Normal University (Permit Number: AEECSDNU2017004).

### Fish rearing

Healthy common carp, *Cyprinus carpio* L. (180 g on average) were obtained from the Fresh Water Fishery Research Institute of Shandong Province. Carp were maintained at 20–25 °C in recirculating water and fed twice a day with commercial feed for more than 2 weeks before experimental use [38–40]. The tissue samples, including foregut, liver, spleen, muscle, hindgut, oral epithelial, head kidney, gill, skin, gonad and brain, were obtained from normal common carp, and separately frozen in liquid nitrogen until further use for RNA extraction [41–43].

To examine the expression profiles of common carp PGRPs during ontogeny, four pairs of parent fish were selected for artificial propagation. Fertilized eggs were incubated in water reservoir at 28–30 °C with sufficient oxygen [1, 44]. After fertilization, 5 replicates of developmental samples were collected at 1, 2, 3, 4, 6, 10, 16, 24 and 36 dpf (days post fertilization) for total RNA isolation [16].

### Bacterial challenges in vivo

*A. hydrophila* used in the study was obtained from China Center for Type Culture Collection and incubated in Luria-Bertani medium at 28 °C overnight under continuous shaking. Adult common carp were anaesthetized and inject intraperitoneally with 5 × 10^7^ cfu/ml formalin-inactivated *A. hydrophila*, while control groups were injected with phosphate buffered saline (PBS, pH 7.4) [16]. At 3 h, 6 h, 12 h, 1d, 2d, 3d and 5d post injection, three individuals in each group were euthanized and sampled for total RNA extraction following the procedures described in previous studies [35, 45]. The fish were euthanatized by immersion in a solution of Tricaine Methane Sulfonate (MS-222, Sigma Aldrich) at a concentration of 100 mg/l of water and sampled, all procedures performed under anesthesia and all efforts were made to minimize fish suffering. The fish after the study were disinfected, sealed and treated according to the guidelines of the protocol approved by the Animal Experimental Ethics Committee of Shandong Normal University (Permit Number: AEECSDNU2017004). The collected tissues includes gill, liver, skin, spleen, hindgut, foregut and head kidney. Total RNA was extracted (Tiangen) and reverse transcribed to cDNA (Tiangen). The isolated RNA was measured by UV-spectrophotometer to determine the concentration and quality. To prepare 1st strand cDNA, 2 μg of total RNA treated with 2 μl gDNase buffer was subjected to reverse transcription in 30 μl reactions by using the FastQuent RT Kit (Tiangen) according to the protocol. Finally, the synthesized cDNA was kept at − 20 °C for further analysis [35, 45].

### Cloning and analysis of *Ccpgrp5* and *Ccpgrp6* cDNA

Amplification of the cDNA fragment of *Ccpgrp5* from the spleen of common carp were performed as follow: the specific primers were designed based on the conserved regions of the sequence of PGRP5 in fish species. PCR amplification was performed with the following condition: 94 °C for 3 min, followed by 32 cycles of 94 °C for 30 s, 60 °C for 30 s, and 72 °C for 40 s. The PCR products were ligated into the pMD18-T vector, then was subsequently transformed into competent *E. coli* DH-5α for sequencing. The full-length of the *Ccpgrp5* were obtained by RACE (rapid amplification of the cDNA ends) using the 3′-full and 5′-full RACE core set (TaKaRa). The primers used are shown in Table 1. Amplification of *Ccpgrp6* was performed following the same protocol as for *Ccpgrp5*. PCR of *Ccpgrp6* was performed with the following condition: 94 °C for 3 min, followed by 32 cycles of 94 °C for 30 s, 60 °C for 30 s, and 72 °C for 60 s. The primers used are shown in Table 1.

**Table 1:** List of primer sequences used in the study

Name	Sequence	Application
PGRP5-F	5′-CCTCAGCTCGCTCACATCCA-3′	cDNA amplification
PGRP5-R	5′-AGAGTATAAATGTTCCCCCGGA-3′	
PGRP6-F	5′-GTGTACACTCTCTCTGGTTTGC-3′	
PGRP6-R	5′-CTGGTAACGCTCCCATGTCTGG-3′	
β-actin-F	5′-TGGCATCACACCTTCTACAAC-3′	
β-actin-R	5′-GCCCATCTCCTTGCTCGAAGTC-3′	
5′RACE Outer primer	5′-CATGGCTACATGCTGACAGCCTA-3′	RACE gene specific primers
5′RACE Inner primer	5′-CGCGGATCCACAGCCTACTGATGATCAGTCGATG-3′	
3′RACE Outer primer	5′-TACCGTCGTTCCACTAGTGATTT-3′	
3′RACE Inner primer	5′-CGCGGATCCTCCACTAGTGATTTCACTATAGG-3′	
5′ PGRP5 Outer primer	5′-TGGATGTGAGCGAGCTGAGGGACG-3′	
5′ PGRP5 Inner primer	5′-ATCACAGTCTGTGCGGGACTCTTC-3′	
3′ PGRP5 Outer primer	5′-CGTCCCTCAGCTCGCTCACATCCA-3′	
3′ PGRP5 Inner primer	5′-GGAGATGGGATGGTGTATGAAGGG-3′	
5′ PGRP6 Outer primer	5′-GTAACTCTTCAGCAGGTCACTCAG-3′	
5′ PGRP6 Inner primer	5′-AACTTCTGACCCGAGAATGAACCC-3′	
3′ PGRP6 Outer primer	5′-CAAGAGTGCGATGGACATGGTGAG-3′	
3′ PGRP6 Inner primer	5′-GTCTTCATCATACTCCTTATACGG-3′	
PGRP5-qF	5′-CCTCAGCTCGCTCACATCCA-3′	Primers for Real-time PCR
PGRP5-qR	5′-CGCCCTTCATACACCATCCC-3′	
PGRP6-qF	5′-ATGGTGAGGTATGACTTC-3′	
PGRP6-qR	5′-CTTGTCTGTGTCCGTATA-3′	
S11-F	5′-CCGTGGGTGACATCGTTACA-3′	
S11-R	5′-TCAGGACATTGAACCTCACTGTCT-3′	

All the primer sequences used in gene clone and Real-time quantitative PCR of *Ccpgrp5, Ccpgrp6* and internal reference gene

The structural domains of CcPGRP5 and CcPGRP6 were analyzed using the SMART (a simple modular architecture research tool) program (http://smart.embl-heidelberg.de/). The amino acid sequence alignment was performed with MegAlign in DNAstar 7.0 using the method of ClustalW. Prediction of theoretical signal peptide and transmembrane domain was conducted using SignalP 4.1 (http://www.cbs.dtu.dk/services/SignalP/). Multiple sequence alignment was conducted using DNAMAN. The phylogenetic tree was generated based on the deduced amino acid sequences using the Neighbour-Joining method with MEGA6.0. All sequences used for the phylogenetic analysis were listed in Table 2.

**Table 2:** Sequences of peptidoglycan recognition protein used for phylogenetic tree construction and multiple sequence alignment

Species	Protein	Accession no.	Species	Protein	Accession no.
*Homo sapiens*	PGRP1	NP_005082	*Drosophila melanogaster*	PGRP-SA	NP_572727
*Homo sapiens*	PGRP2	NP_443122	*Drosophila melanogaster*	PGRP-SC1A	NP_610407
*Homo sapiens*	PGRP3	NP_443123	*Drosophila melanogaster*	PGRP-SD	NP_610410
*Homo sapiens*	PGRP4	NP_065126	*Drosophila melanogaster*	PGRP-LA	NP_996028
*Mus musculus*	PGRP1	NP_033428	*Drosophila melanogaster*	PGRP-LB	NP-731575
*Mus musculus*	PGRP2	NP_067294	*Drosophila melanogaster*	PGRP-LC	NP_729468
*Mus musculus*	PGRP3	NP_997130	*Drosophila melanogaster*	PGRP-LD	NP_001027113
*Mus musculus*	PGRP4	NP_997146	*Drosophila melanogaster*	PGRP-LE	NP-573078
*Danio rerio*	PGRP2	NP_001038631	*Drosophila melanogaster*	PGRP-LF	NP_648299
*Danio rerio*	PGRP5	NP_001037786	*Sebastes schlegeli*	PGRP-L2	GU126381
*Danio rerio*	PGRP6	NP_001038687	*Argopecten irradians*	PGRP	AAR92030
*Chlamys farreri*	PGRP-S1	AAY53765	*Euprymna scolopes*	PGRP1	AAY27973
*Asterias rubens*	PGRP- S1a	ABB04459	*Euprymna scolopes*	PGRP2	AAY27974
*Asterias rubens*	PGRP- S2a	ABB04460	*Euprymna scolopes*	PGRP3	AAY27975
*Canislupus familiaris*	PGRP2	XP-852999	*Euprymna scolopes*	PGRP4	AAY27976
*Sus scrofa*	PGRP1	NP-001001260	*Ornithorhynchusanatinus*	PGRP-L	XP-001506175
*Sus scrofa*	PGRP-L	NP-998903	*Ornithorhynchusanatinus*	PGRP2	XP-001520922
*Gallus gallus*	PGRP2	NP-001038151	*Bos Taurus*	PGRP1	AAL87002
*Equus caballus*	PGRP4	XP-001494309	*Bos Taurus*	PGRP2	DAA19524
*Sciaenop ocellatus*	PGRP2	ACJ13032	*Bos Taurus*	PGRP3	XP-611696
*Tetraodonnigroviridis*	PGRP-L	CAG06114	*Bos Taurus*	PGRP4	XP-874055
*Chlamys farreri*	PGRP-S1	AAY53765	*Macaca mulatta*	PGRP1	XP_001103121
*Ctenopharyngodon idella*	PGRP5	AFE48096	*Macaca mulatta*	PGRP3	XP_001110242
*Ctenopharyngodon idella*	PGRP6	ADL41186	*Rattus norvegicus*	PGRP1	AAF73252
*Camelus dromedarius*	PGRP1	CAC84130	*Rattus norvegicus*	PGRP3	XP_008759474
*Xenopus tropicalis*	PGRP1	NP_001015775	*Trichoplusia ni*	PGRP	O76537
*Xenopus tropicalis*	PGRP2	ABO15681	*Anoplopoma fimbria*	PGRP-SC2	ACQ58764
*Oreochromis niloticus*	PGRP-SC2	XP_003441739	*Azumapecten farreri*	PGRP-S1	AAY53765
*Saimo rerio*	PGRP-S	NP-001037786	*Sebastes schlegelii*	PGRP-L	ADC93708
*Pongo abelii*	PGRP1	XP-002829481	*Monodelphis domestica*	PGRP1	XP-001363587
*Monodelphis domestica*	PGRP3	XP-007481978	*Salmo salar*	PGRP-S	BT049722

### Real-time quantitative PCR

*Real-time quantitative* PCR was performed with a LightCycler 96 Real-Time PCR System (Roche) using SYBR Green Real Master Mix (Tiangen). General cycling conditions were set to: incubation at 95 °C for 3 min, followed by 40 cycles for 10 s at 95 °C, 30 s at 62 °C and 20 s at 72 °C. The comparison between the internal reference gene 40S ribosomal protein S11 and β-actin by *BestKeeper* gave decreasing ranking order for S11 and β-actin (http://150.216.56.64/referencegene.php?type=reference) [46]. Expression of mRNA was normalized with the expression of 40S ribosomal protein S11 in each sample. The Relative expression was determined using the 2^(-ΔΔCt)^ method [39, 47]. The primers for *Ccpgrp5* and *Ccpgrp6* are shown in Tables 1 and 2 respectively. The efficiencies of each primers for *Ccpgrp5*, *Ccpgrp6* and internal reference gene S11 were 95.78, 100.67 and 99.43% respectively. Each PCR was performed with triplicate samples.

### Statistical analysis

The significance of the average fold change between the challenged group and the control group were analyzed using the Graphpad Prism 6. The significant differences were considered at *p* < 0.05. A two-way analysis of variance (ANOVA) was performed to test differences in gene expression in each tissue.

## Abbreviations

ANOVA: Two-way analysis of variance
DAP: Diaminopimelic acid
dpf: Days post fertilization
Imd: Immune deficiency signal transduction pathways
LPS: Lipopolysaccharide
MurNAc: N-acetylmuramic acid
ORF: Open reading frame
PAMPs: Pathogen-associated molecular patterns
PGLYRP-2 (PGLYRP-1, PGLYRP-3, PGLYRP-4): Peptidoglycan recognition proteins
PGRP: Peptigoglycan recognition protein
PI: Isoelectric point
proPO: Prophenoloxidase
PRRs: Pattern recognition receptors
RACE: Rapid amplification of the cDNA ends
SDS-PAGE: Sodium dodecyl sulfate polyacrylamide gel electropheresis
SMART: A simple modular architecture research tool
UTR: Untranslated region
